# Supplementary material for: Extended haplotype-phasing of long-read de novo genome assemblies using Hi-C
Source: Nat Commun. 2021 Apr 28;12:1935. doi: 10.1038/s41467-020-20536-y (PMC8081726; doi:10.1038/s41467-020-20536-y)
Supplement: Supplementary file 1 — Supplementary Information [file 41467_2020_20536_MOESM1_ESM.pdf]

Supplementary Information, Extended haplotype-phasing of long-read *de novo* genome assemblies using Hi-C, Kronenberg et al.

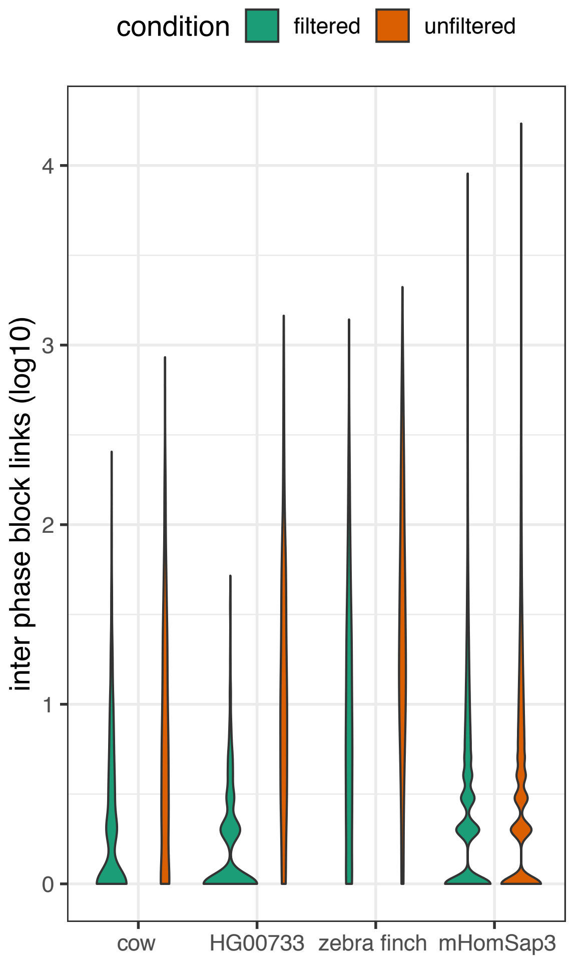

Supplementary Figure 1. Hi-C links between phase blocks within primary contigs. Each violin plot shows the log10 distribution of Hi-C links between phase blocks before and after map quality filtering. These counts are restricted to links within primary contigs. Zero counts are not shown. The shape of this distribution is affected by an interaction between the length of long-range Hi-C contacts and the heterozygosity level.

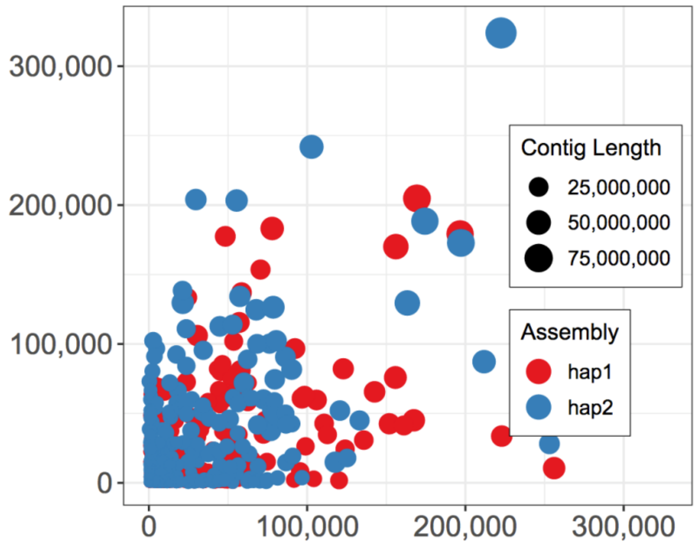

Supplementary Figure 2. Phasing for HG00733 supernova contigs (GCA_002022865.1). Markers from mother are on the x-axis, father on the y-axis. Contig size is indicated by size of the data point.

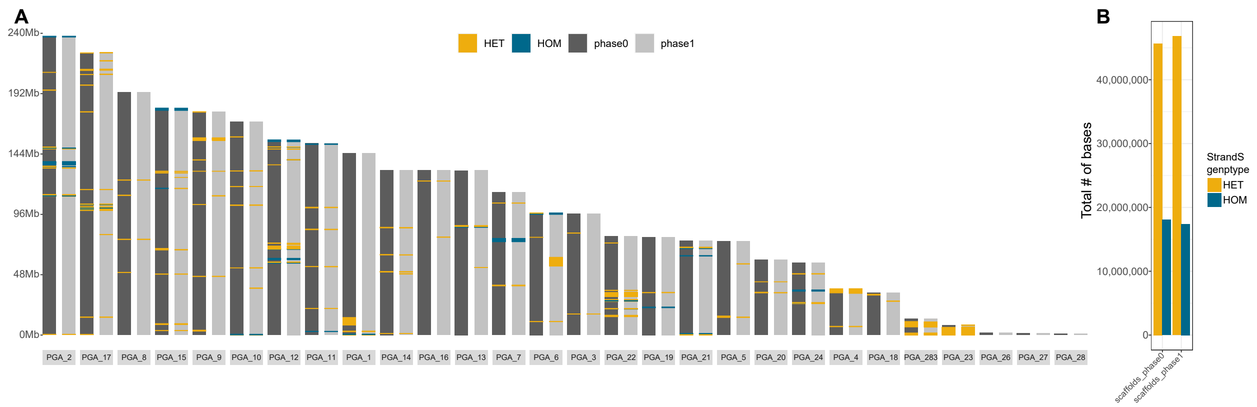

Supplementary Figure 3. Concordance of Strand-seq data and FALCON-Phase HG00733 scaffolds.

a, Both phased scaffolds are shown for each chromosome in dark and light gray bars, respectively. Homozygous differences between the Strand-seq data and our scaffolds (HOM - blue) suggest scaffold misorientations or putative inversions. Heterozygous differences (HET - yellow) in scaffold directionality highlight putative heterozygous inversions, chimeric sequence, collapsed duplicated sequence, or satellite DNA. **b**, The total number of bases genotyped in Strand-seq data as homozygous (HOM - blue) or heterozygous (HET - yellow).

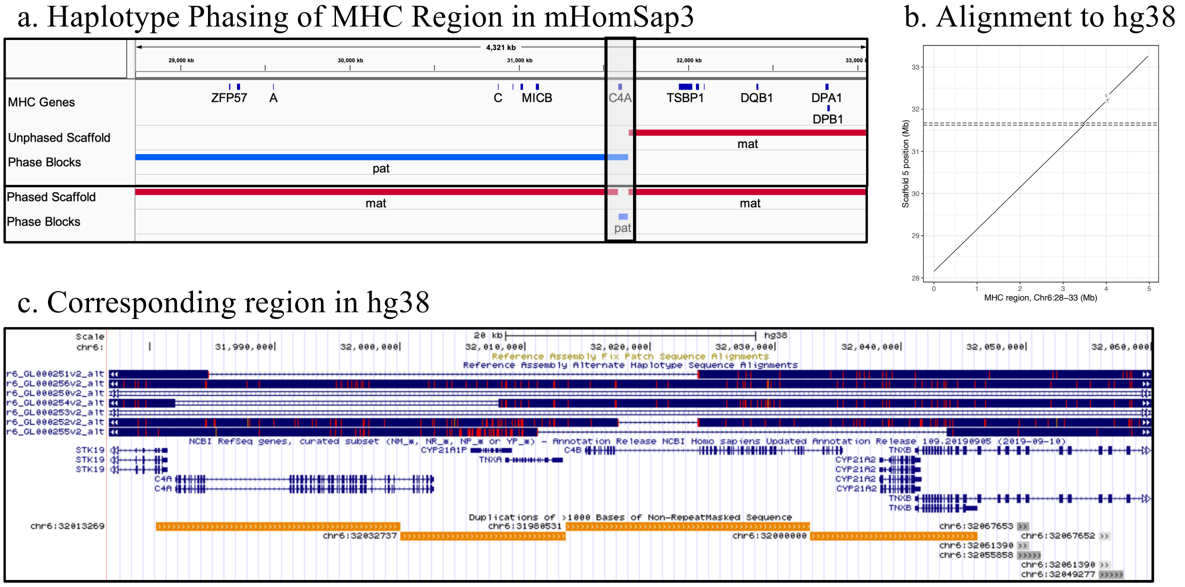

Supplementary Figure 4. Phasing across MHC in mHomSap3. a, Haplotype phase blocks were called using Merqury[1] before (above) and after (below) FALCON-Phase was run on the scaffolds. In the unphased scaffolds, the MHC region is covered by two large phased blocks (paternal phase block length 3,009,944, maternal phase block length 1,455,202). A phase switch error near the CA4 gene in the original scaffold was corrected with FALCON-Phase and the resulting scaffold is primarily the maternal haplotype. A small (53,107 bp) block of paternal haplotype remains in the CA4 region. **b,** Alignment of this region to chromosome 6 in the human reference reveals that the phasing error overlaps a structural difference with the hg38 reference. **c,** Annotations of the region containing the phasing error in mHomSap3 in hg38 show segmental duplications and numerous alternate alleles.

Supplementary Table 1. FALCON-Unzip contig summary statistics

Sample	Zebra Finch	Cow	HG00733	mHomSap3
PacBio Data (Coverage)	83.5Gb (70X)	275 Gb (100X)	263 Gb (90X)	174 Gb (62X)
Primary Contig N	1,941	1,427	865	1,069
Primary Contig Length (Gb)	1.15	2.71	2.89	2.88
Primary Contig N50 (Mb)	2.93	31.4	27.8	22.4
Haplotig N	4,657	5,879	7,863	6,728
Haplotig L (Gb)	0.856	2.45	2.43	2.34
Haplotig N50 (Mb)	0.344	2.48	0.567	0.672

Supplementary Table 2. Hi-C mate pair mapping statistics

Sample	Zebra Finch	Cow	HG00733	mHomSap3
Total Read Pairs	625 M	395 M	1006 M	1686 M
Filtered Pairs (% total reads)	275 M (44.1%)	64.5M (16.3%)	128 M (12.7%)	186 M (11.0%)
Map Dist > 10 kb (% filt reads)	205 M (74.4%)	53 M (82.2%)	91 M (71.2%)	126 M (68.1%)
Map Dist > 50 kb (% filt reads)	163 M (59.1%)	46 M (70.6%)	65 M (50.4%)	96 M (51.6%)
Map Dist > 100 kb (% filt reads)	7.8 M (2.85%)	14 M (22.4%)	4.5 M (3.51%)	12 M (6.56%)

Supplementary Table 3. Contig phasing accuracy on HG002 HiFi dataset.

**Sample**	**HG002**
Heterozygosity measured with k-mers (%)	0.293
Primary Assembly Length (Gb)	3.03
Primary Contig N50 (Mb)	32.1
Mean Phase Block Length (kb)	118
Proportion of Genome Unzipped (%)	61.3 %
IPA Primary Contig Accuracy (%)	76.0 %
IPA Haplotig Accuracy (%)	97.6 %
FALCON-Phase Contig Accuracy (%)	82.7 %
PacBio HiFi Data	35X (15 kb and 20 kb libraries)
Hi-C Data	69X of 2x250bp (442M read pairs)
Assembler	IPA[2] v1.1.2, default parameters

Supplementary Table 4. Comparison of FALCON-Phase to Strand-seq based assembly method.

Method	Sample	Switch Error Rate		Hamming Distance	
		Phase 0	Phase 1	Phase 0	Phase 1
FALCON-Phase	HG00733	0.781 %	0.782 %	36.9 %	36.9 %
Porubsky et al. v1.1	HG00733	0.390 %	0.395 %	1.01 %	0.993 %

References

1. Rhie, A., Walenz, B. P., Koren, S. & Phillippy, A. M. Merqury: Reference-free quality, completeness, and phasing assessment for genome assemblies. *Genome Biol.* (2020). doi:10.1186/s13059-020-02134-9
2. Kronenberg, Z. IPA HiFi Genome Assembler. Available at: https://github.com/PacificBiosciences/pbipa. (Accessed: 7th October 2020)
