# Supplementary material for: Extended haplotype-phasing of long-read de novo genome assemblies using Hi-C
Source: Nat Commun. 2021 Apr 28;12:1935. doi: 10.1038/s41467-020-20536-y (PMC8081726; doi:10.1038/s41467-020-20536-y)
Supplement: Supplementary file 3 — Reporting Summary [file 41467_2020_20536_MOESM3_ESM.pdf]

nature research

Corresponding author(s): Zev Kronenberg, Sarah Kingan

Last updated by author(s): Nov 8, 2020

Reporting Summary

Nature Research wishes to improve the reproducibility of the work that we publish. This form provides structure for consistency and transparency in reporting. For further information on Nature Research policies, see our Editorial Policies and the Editorial Policy Checklist.

Statistics

For all statistical analyses, confirm that the following items are present in the figure legend, table legend, main text, or Methods section.

n/a	Confirmed	
☒	☐	The exact sample size (n) for each experimental group/condition, given as a discrete number and unit of measurement
☒	☐	A statement on whether measurements were taken from distinct samples or whether the same sample was measured repeatedly
☒	☐	The statistical test(s) used AND whether they are one- or two-sided<br>*Only common tests should be described solely by name; describe more complex techniques in the Methods section.*
☒	☐	A description of all covariates tested
☒	☐	A description of any assumptions or corrections, such as tests of normality and adjustment for multiple comparisons
☒	☐	A full description of the statistical parameters including central tendency (e.g. means) or other basic estimates (e.g. regression coefficient) AND variation (e.g. standard deviation) or associated estimates of uncertainty (e.g. confidence intervals)
☒	☐	For null hypothesis testing, the test statistic (e.g. F, t, r) with confidence intervals, effect sizes, degrees of freedom and P value noted<br>*Give P values as exact values whenever suitable.*
☒	☐	For Bayesian analysis, information on the choice of priors and Markov chain Monte Carlo settings
☒	☐	For hierarchical and complex designs, identification of the appropriate level for tests and full reporting of outcomes
☒	☐	Estimates of effect sizes (e.g. Cohen's d, Pearson's r), indicating how they were calculated

Our web collection on statistics for biologists contains articles on many of the points above.

Software and code

Policy information about availability of computer code

Data collection	no software was used in data collection
Data analysis	FALCON-Phase (May 18 2018), bwa v 0.7.17, bedtools v 2.27.1, samtools v 1.7, mummer v 4.0.0, snakemake v4.8, FALCON-Unzip Aug 13 2018 & pbbiocona v 0.0.2 and v0.0.6, SAMBLASTER41 (commit 37142b37e4f0026e1b83ca3f1545d1807ef77617), Proximo Hi-C (commit 145c01be162be85c060c567d576bb4786496c032, Juicebox v1.8.8, meryl in canu 1.7, Genomescope 1.0, mummer v 3.2.3, D-Genies (2017), minimap2.14 and v2.17, purge haplotigs July 2018, breakpointR v3.12

For manuscripts utilizing custom algorithms or software that are central to the research but not yet described in published literature, software must be made available to editors and reviewers. We strongly encourage code deposition in a community repository (e.g. GitHub). See the Nature Research guidelines for submitting code & software for further information.

Data

Policy information about availability of data

All manuscripts must include a data availability statement. This statement should provide the following information, where applicable:

- Accession codes, unique identifiers, or web links for publicly available datasets
- A list of figures that have associated raw data
- A description of any restrictions on data availability

Zebra finch PacBio long reads, Hi-C data, parental short read data, triobinned parental Canu assemblies: [https://vgp.github.io/genomeark/Taeniopygia_guttata/]. FALCON-Unzip contigs: [https://www.ncbi.nlm.nih.gov/bioproject/PRJNA604785], [https://www.ncbi.nlm.nih.gov/bioproject/PRJNA604786]. FALCON-Phase contigs: [https://www.ncbi.nlm.nih.gov/bioproject/PRJNA604789], [https://www.ncbi.nlm.nih.gov/bioproject/PRJNA604788]. FALCON-Phase scaffolds: [https://www.ncbi.nlm.nih.gov/bioproject/PRJNA604793], [https://www.ncbi.nlm.nih.gov/bioproject/PRJNA604794].

Cow PacBio long reads, Hi-C data, parental short read data, triobinned parental canu assemblies: [https://www.ncbi.nlm.nih.gov/bioproject/PRJNA432857]. FALCON-Unzip contigs: [https://www.ncbi.nlm.nih.gov/bioproject/PRJNA604814], [https://www.ncbi.nlm.nih.gov/bioproject/PRJNA604813]. FALCON-Phase contigs: [https://www.ncbi.nlm.nih.gov/bioproject/PRJNA604823], [https://www.ncbi.nlm.nih.gov/bioproject/PRJNA604824]. FALCON-Phase scaffolds: [https://www.ncbi.nlm.nih.gov/bioproject/PRJNA604826], [https://www.ncbi.nlm.nih.gov/bioproject/PRJNA604827].

HG00733 PacBio long reads: [https://www.ncbi.nlm.nih.gov/sra/SRR7615963]. Hi-C data: [https://www.ncbi.nlm.nih.gov/sra/ERR1225141] – [https://www.ncbi.nlm.nih.gov/sra/ERR1225146]. Parental short read data: [https://www.ncbi.nlm.nih.gov/bioproject/PRJNA42573]. Triobinned parental canu assemblies: [https://obj.umiacs.umd.edu/marbl_publications/triobinning/h_sapiens_HG00733_dad.fasta], [https://obj.umiacs.umd.edu/marbl_publications/triobinning/h_sapiens_HG00733_mom.fasta]. FALCON-Unzip contigs: [https://www.ncbi.nlm.nih.gov/bioproject/PRJNA604844], [https://www.ncbi.nlm.nih.gov/bioproject/PRJNA604843]. FALCON-Phase contigs: [https://www.ncbi.nlm.nih.gov/bioproject/PRJNA604845], [https://www.ncbi.nlm.nih.gov/bioproject/PRJNA604846]. FALCON-Phase scaffolds: [https://www.ncbi.nlm.nih.gov/assembly/GCA_003634875.1]

mHomSap3 PacBio long reads, Hi-C data, parental short read data: [https://vgp.github.io/genomeark/Homo_sapiens/]. Triobinned parental canu assemblies: [https://genomeark.s3.amazonaws.com/species/Homo_sapiens/mHomSap3/assembly_nhgri_trio_1.6/intermediates/mHomSap3_mat_t1.fasta.gz], [https://genomeark.s3.amazonaws.com/species/Homo_sapiens/mHomSap3/assembly_nhgri_trio_1.6/intermediates/mHomSap3_pat_t1.fasta.gz]. FALCON-Unzip contigs: [https://www.ncbi.nlm.nih.gov/bioproject/PRJNA604831], [https://www.ncbi.nlm.nih.gov/bioproject/PRJNA604832]. FALCON-Phase contigs: [https://www.ncbi.nlm.nih.gov/bioproject/PRJNA604836], [https://www.ncbi.nlm.nih.gov/bioproject/PRJNA604835]. FALCON-Phase scaffolds: [https://www.ncbi.nlm.nih.gov/bioproject/PRJNA604839], [https://www.ncbi.nlm.nih.gov/bioproject/PRJNA604838].

HG002 PacBio HiFi Reads: [https://www.ncbi.nlm.nih.gov/sra/SRR10382244], [https://www.ncbi.nlm.nih.gov/sra/SRR10382245], [https://www.ncbi.nlm.nih.gov/sra/SRR10382248], [https://www.ncbi.nlm.nih.gov/sra/SRR10382249]. Hi-C data: [https://github.com/human-pangenomics/HG002_Data_Freeze_v1.0]. Parental short read data: [ftp://ftp-trace.ncbi.nlm.nih.gov/giab/ftp/data/AshkenazimTrio/HG004_NA24143_mother/NIST_Illumina_2x250bps/reads/], [ftp://ftp-

trace.ncbi.nlm.nih.gov/giab/ftp/data/AshkenazimTrio/HG003_NA24149_father/NIST_Illumina_2x250bps/reads/]. IPA contigs:[https://www.ncbi.nlm.nih.gov/bioproject/PRJNA667512], [https://www.ncbi.nlm.nih.gov/bioproject/PRJNA667511]. FALCON-Phase contigs: [https://www.ncbi.nlm.nih.gov/bioproject/PRJNA667513], [https://www.ncbi.nlm.nih.gov/bioproject/PRJNA667514].

Field-specific reporting

Please select the one below that is the best fit for your research. If you are not sure, read the appropriate sections before making your selection.

[x] Life sciences [ ] Behavioural & social sciences [ ] Ecological, evolutionary & environmental sciences

For a reference copy of the document with all sections, see nature.com/documents/nr-reporting-summary-flat.pdf

Life sciences study design

All studies must disclose on these points even when the disclosure is negative.

Sample size	4 species, 1 trio each
Data exclusions	none
Replication	not applicable
Randomization	not applicable
Blinding	not applicable

Reporting for specific materials, systems and methods

We require information from authors about some types of materials, experimental systems and methods used in many studies. Here, indicate whether each material, system or method listed is relevant to your study. If you are not sure if a list item applies to your research, read the appropriate section before selecting a response.

Materials & experimental systems

n/a	Involved in the study	
[x]	[ ]	Antibodies
[x]	[ ]	Eukaryotic cell lines
[x]	[ ]	Palaeontology and archaeology
[x]	[ ]	Animals and other organisms
[x]	[ ]	Human research participants
[x]	[ ]	Clinical data
[x]	[ ]	Dual use research of concern

Methods

n/a	Involved in the study	
[x]	[ ]	ChIP-seq
[x]	[ ]	Flow cytometry
[x]	[ ]	MRI-based neuroimaging
